# Supporting identity and relationships amongst people with dementia through the use of technology: a qualitative interview study

**DOI:** 10.1080/17482631.2021.1920349

**Published:** 2021-05-06

**Authors:** Gemma Goodall, Lara André, Kristin Taraldsen, J Artur Serrano

**Affiliations:** a Department of Neuromedicine and Movement Science, Norwegian University of Science and Technology, NTNU, Trondheim, Norway; b Departamento de Ação Social e Saúde (Department of Social Action and Health), Santa Casa Da Misericórdia De Lisboa (SCML), Lisbon, Portugal; c Norwegian Centre for eHealth Research, University Hospital of North Norway, Tromsø, Norway

**Keywords:** Dementia, technology, narrative, identity, relationships, symbolic interactionism, reflexive thematic analysis, qualitative research methods

## Abstract

Meaningful activities in dementia care can promote the co-construction of narrative identity in caregiving relationships, helping to preserve the sense of self in people with dementia.

**Purpose**: Informed by symbolic interactionism and Deweyan transactionalism, the aim of this study was to develop a transactional model of how narrative identity and relationships are promoted through the use of a new technological solution, SENSE-GARDEN, that uses digital technologies and multisensory stimuli to facilitate individualized, meaningful activities.

**Method**: We conducted a qualitative interview study to explore the experiences of people with moderate to advanced dementia and their caregivers in Norway and Portugal. After using SENSE-GARDEN for 12–16 weeks, 20 participants (7 persons with dementia and 13 caregivers) were interviewed. The interviews were analysed using reflexive thematic analysis.

**Results**: Three themes were generated: openness, learning, and connection. Findings suggest that SENSE-GARDEN can stimulate emotional experiences, preserve narrative identity, and foster interpersonal relationships. These findings are illustrated through a transactional model.

**Conclusion**: This study highlights the complex multitude of factors affecting person-environment interactions in which narrative identity and relationships are constructed. To better understand these factors, future work should adopt a holistic approach to studying new methods of creating meaningful activities in dementia care.

## Introduction

Common forms of dementia, such as Alzheimer’s Disease, primarily affect memory, language, and behaviour. These impacts can lead to difficulties in communicating and socializing with others, which means people living with dementia often experience stigmatization as a result of behaving in a way that deviates from social norms (Mukadam & Livingston, 2012). The stigma surrounding dementia is characterized by negative perceptions of the disease, particularly with regard to the loss of self. For instance, as Beard et al. (2009) note, one dominant story that has been portrayed about people with dementia is that their talk is meaningless, their recollections are of little importance, and their memories are defective. However, the importance of challenging these perceptions and advocating positive attitudes towards dementia, in both social and scientific contexts, is becoming increasingly recognized (Swaffer, 2014; Zeilig, 2014; Dementia Engagement and Empowerment Project, 2014; Brotherhood et al., 2017). Discourse on dementia is shifting away from a reductionist biomedical perspective, that portrays the disease primarily in terms of loss, towards a more holistic perspective that considers the person with dementia not as a “sufferer” but as an individual who can be supported to cope and potentially live well with the condition (Beard et al., 2009; Kitwood, 1997; Swaffer, 2014). According to sociological perspectives, the loss of self that is experienced by people with dementia has much to do with the attitudes of others, rather than being caused solely by the disease itself (Sabat & Harré, 1992; Surr, 2006). As such, the way in which people with dementia experience social interactions in care impacts not only their sense of self but also their state of psychological well-being (Clare et al., 2008; Lee, Boltz, Lee & Algase, 2017).

Recently, international policymakers have suggested that people with dementia often receive sub-optimal care, and there is a need to understand how to deliver high-quality care particularly to those in later stages of the disease (OECD Policy Brief, 2018; World Dementia Council, 2018). This echoes previous research that has voiced these issues, with Baldwin (2008) labelling care homes as places in which residents with dementia “are essentially warehoused until death”. Baldwin (2008) also called for the development of services that encourage opportunities for expression and co-construction of narrative in care institutions. The role of others has been well established in maintaining a sense of identity amongst people living with dementia (Kitwood, 1997; Mills, 1997; Surr, 2006), and as such, there is a need to consider how care homes can be places in which others are given the tools and opportunities to co-construct the narratives of residents with dementia. In Norway, the Government acknowledges the lack of meaningful activities for people living with dementia, and states that future care services need to be oriented towards the individual’s wishes, interests, and habits (Norwegian Ministry of Health and Care Services, 2015).

### Technologies for reminiscence

Technology has much to offer in supporting, facilitating, and creating new possibilities for meaningful activities in dementia care that promote a sense of identity. Digital technologies, such as mobile and tablet apps, can support collaborative explorations of life events by people with dementia and caregivers, encouraging the caregiver to reflect and learn more about the individual (Maiden et al., 2013). These technologies can also be used as a means of conveying the narrative of people living with dementia. Purves et al. (2011) suggest that the use of photographs, films, and music can bring history to life, extending the reach of stories to others, not only to the person with dementia.

With the rapid development of technological solutions for care, there has been an increasing amount of reminiscence technologies developed for people living with dementia. One popular area of study is the use of digital life books, which are often mobile or tablet apps that combine music, photographs, videos, and narration to create personalized content for people with dementia and their family members (Critten & Kucirkova, 2019; Hashim et al., 2015; Laird et al., 2018; Ryan et al., 2018). Studies of digital life books suggest that they can encourage the delivery of person-centred care amongst staff and improve quality of life and autobiographical memory in people with dementia (Subramaniam & Woods, 2016). Even in later stages of dementia, digital story apps can help to support a sense of self-identity and empowerment amongst individuals (Critten & Kucirkova, 2019; Park et al., 2017).

These types of apps are also becoming readily accessible on a commercial level. For instance, *Book of You* is a digital reminiscence book that can be purchased for individuals with dementia and their family members (Book of You, 2021). *Book of You* is also available for care organizations to buy, which includes not only the digital books for residents, but training for staff members. Another example is *Storii*, a free online resource that allows families to create an interactive, multimedia life biography with the use of photos, music, videos, audio recordings and text (Storri, 2021).

Whilst digital apps and multimedia biographies have been shown to be effective in dementia care, more immersive approaches to life story work are currently being explored. For example, virtual reality can be a means of providing individuals with dementia the opportunity to interact with locations and events when it is no longer possible to do so in-person. For instance, Hodge et al. (2018) explored the use of virtual reality experiences for people with dementia, designing various environments including a personalized virtual reality experience of a concert venue for one couple in particular wherein the wife had dementia. Participants were able to engage in new experiences, which served as a talking point amongst couples. However, the authors identified potential barriers to use such as some participants feeling “silly” whilst wearing the headset and the headset being too heavy to wear. Additionally, whilst caregivers expressed that they enjoyed watching their relatives interact with the virtual environment, they wished they could have joined them in some way.

The importance of providing technologies that can be used as a joint activity is supported by research in this field. Laird et al. (2018) found that a reminiscence iPad app (InspireD) significantly improved the quality of carer and patient relationship as well as mutuality and subjective well-being amongst people with dementia and their family members. Other studies suggest that personalized digital media can be used as a tool for starting conversations and supporting interaction (Davis and Shenk, 2015; Hashim et al., 2015; Samuelsson & Ekström, 2019). As such, it is important to identify ways of creating an immersive environment whilst still providing the opportunity for social interaction. Furthermore, it has been suggested that providing dedicated spaces in nursing homes for private interaction is vital for maintaining relationships and enabling connections between spouses with partners living with dementia in long-term care (Førsund & Ytrehus, 2018). Therefore, providing not just the technology but a space in which residents, family, and staff can share private and meaningful interactions may result in benefits for all users.

### SENSE-GARDEN

The SENSE-GARDEN is a novel, technological solution used to deliver an individualized intervention (i.e., the SENSE-GARDEN intervention) to people with moderate to severe dementia. It was developed as part of an interdisciplinary EU project (SENSE-GARDEN, 2021) that aimed to create individualized, immersive spaces for people living with dementia in Belgium, Norway, Portugal, and Romania. A SENSE-GARDEN is a room built inside of a dementia care environment (i.e., care home or hospital) that combines immersive technologies, digital media, and multisensory stimuli to create environments personalized to the life story of the person with dementia. The concept builds upon techniques from reminiscence therapy, in which the individual is encouraged to remember and reflect upon people, places, and events from their lives (Butler, 1963). By using digital technologies to present familiar music, photographs, films and scents within an immersive environment, it is hoped that the SENSE-GARDEN can provide staff and residents with dementia new opportunities to engage with the life story of the individual.

Whilst there has been research on the combination of digital and multisensory environments (see Moyle et al., 2018 *Virtual Reality Forest*, for example), there has been little work conducted on creating immersive, multisensory environments tailored to the life story of the individual with dementia. To date, *Snoezelen* has been the most widely used approach to using immersive, sensory stimulation with people living with dementia (Pinto et al., 2020). Deriving from the Dutch terms *snuffelen* (to seek and explore) and *doezelen* (to relax), *Snoezelen* multisensory environments offer a choice of olfactory, auditory, visual and/or tactile stimuli to individuals so that they may explore the stimuli whilst being in a state of relaxation (Baker et al., 2001). However, *Snoezelen* environments are not used for reminiscence purposes. SENSE-GARDEN has a different approach in that it aims at engaging the person with dementia in reminiscence activity through the use of personalized stimuli that is based on the life story of the individual. The use of innovative technology means that the stimuli can be adjusted to the individual, and thus, every SENSE-GARDEN session is unique to each user. Through presenting personalized content in a multisensory way, the person with dementia is immersed in their own life story.

Previous studies on SENSE-GARDEN have included the exploration of initial user perspectives towards the overall concept (Goodall et al., 2019a) and care staff experiences of the space in a Norwegian care home (Goodall, Taraldsen, Granbo et al., 2020). However, the experiences of people with dementia and their family members have yet to be explored. Additionally, although digital technologies are being increasingly used in an individualized manner to complement approaches such as life story work and reminiscence therapy for people living with dementia (Goodall, Taraldsen, Serrano et al., 2020), most of the work has been conducted in the homes of people with dementia. There is a need to investigate the use of technology in long-term residential care, also for people living with moderate to severe dementia.

### Aims

The primary aim of this study is to create a transactional model of how narrative identity and relationships are promoted through the use of SENSE-GARDEN. We will address the following research questions: 1) What are the experiences of people with dementia and their caregivers with the new technological intervention, SENSE-GARDEN?, 2) How are narrative identities constructed and shared using SENSE-GARDEN?, and 3) How does SENSE-GARDEN facilitate interactions and communication between people with dementia and caregivers?

## Theoretical positioning: symbolic interactionism and Deweyan transactionalism

### Symbolic Interactionism

The SENSE-GARDEN a) uses meaningful stimuli significant to the individual and b) aims to facilitate meaningful experiences in the present moment. Therefore, this study draws upon symbolic interactionism for the ways in which it considers how individuals interact with one another reciprocally to form meaning (Blumer, 1986). Deriving from George Mead’s (1934) belief that an individual’s sense of self is developed through social interaction with others, symbolic interactionism is a theory that seeks to explain social behaviour in terms of the way people reciprocally interact with each other through symbols. Symbols—such as language, signs, and gestures—may hold different meanings for different people and, as such, will influence how an interaction is interpreted and experienced. The theory was refined and developed by Mead’s student, Herbert Blumer, who described three key premises on which symbolic interactionism is built (Blumer, 1986, p. 2). First, the ways in which an individual behaves towards objects and other individuals is based on personal meanings that the individual has given to them. Second, the meaning of these objects is based on the social interaction that the individual has with others and with society as a whole. Third, these meanings are handled in, and modified through, an interpretive process. In other words, our meaning of the world around us constantly changes through the influence of social interactions and personal experiences.

Previous work in this area has also used symbolic interactionism to provide insights into interpersonal relationships, communication, and couple well-being in dementia care (Hayes et al., 2009; McGovern, 2010; Walmsley & McCormack, 2014). Johnson et al. (2017) used a symbolic interactionist perspective to outline the ways in which caregivers can communicate with people living with advanced dementia. The authors suggest that by interacting with the individual with dementia on a symbolic level, e.g., using photos, expressions and gestures, powerful connections can be made.

### Transactionalism

Given the complexity of the SENSE-GARDEN space, the multi-dimensional nature of narratives, and the intricacy of interpersonal relationships, it is important to go beyond interactions between persons and also consider the wider environment as a whole. As such, this study is also informed by Dewey’s transactional theory, which is concerned with the dynamic nature of person-environment experiences. He writes, “Everything that exists in far as it is known and knowable is in interaction with other things. It is associated, as well as solitary, single.” (Dewey, 1929, p. 175). In other words, individual components of an environment interact with each other in ways that form an overall relationship. In the context of this study, it could be insightful to consider the ways in which the users within SENSE-GARDEN not only reciprocally interact with one another but also with the multisensory stimuli and digital media surrounding them.

One field in which Deweyan transactional perspectives is being increasingly used is that of occupational science (Garrison, 2002; Cutchin, 2004; Dickie et al., 2006; Cutchin & Dickie, 2012; Lavelley, 2017). In adopting Dewey’s holistic approach to person–environment interactions, scholars in this area consider client and practitioner as reflexive social selves (Cutchin, 2004), and imply that occupational practice has much to benefit from considering how occupation is a mode through which individuals function in their “complex totality” (Dickie et al., 2006).

To date, and to our knowledge, the only research on dementia that explicitly refers to Dewey’s transactional theory is a study on the unfolding transactions of assistive technology use amongst people living with dementia and their significant others (Rosenberg & Nygård, 2012). Findings suggested that assistive technology use was influenced by a number of factors including the choice of problem that the technology was meant to address, the user’s experiences and views of the situation, views on how and when the technology should be used, and—most prominently—the view of the individual who had the most power in the decision-making. From these insights, the authors concluded that flexibility and a process-oriented approach are key issues when introducing and prescribing assistive technology to people with dementia (Rosenberg & Nygård, 2012). By applying this theory to the context of SENSE-GARDEN use, future implications may be made for the facilitation and evaluation of similar interventions and technological solutions in dementia care.

Furthermore, theories such as transactionalism have been recognized as useful in the transdisciplinary development of assistive technologies, including technologies for people with dementia. Boger et al. (2017) suggest that dynamic and transactional philosophies that acknowledge the complexity of an individual’s interaction with their environment can help transdisciplinary collaborators in creating technologies that complement the needs, preferences, abilities, and resources of users.

## Methods

### Study design

This study adopted a qualitative interview study design and was part of the SENSE-GARDEN multisite trial (Goodall et al., 2019b). The trial was suspended in March 2020 due to the coronavirus pandemic, and as such, we only included persons with dementia and caregivers who had finished their time in the SENSE-GARDEN study at the time of suspension. Participants had visited the SENSE-GARDEN 2–3 times per week for 16 weeks or 2–3 times per week for 12 weeks. After these visits, 20 participants (7 people with dementia and 13 caregivers) were interviewed. A mixture of individual interviews and group interviews was used, meaning that there were 16 interviews in total (12 individual interviews and 4 group interviews).

Qualitative research is focused not on finding truth but is instead focused on meaning and meaning-making, in which the stories of participants and phenomena can be portrayed (Braun & Clarke, 2019). This resonates with the theory in which this study is rooted, with Deweyan philosophy aiming to seek meaning and knowledge that may make the world a better place. As Cutchin and Dickie (2013) comment, Dewey’s transactional perspective may not solve problems theoretically or practically, but it offers a method of inquiry that can be used to make a better world (p. 9). In adopting a transactional perspective, this study approaches the participants’ reflections and interpretations of their experiences within SENSE-GARDEN in a way that may inform how interventions of this kind can be best optimized to improve the lives of people living with dementia.

### Settings

**Figure 1.**
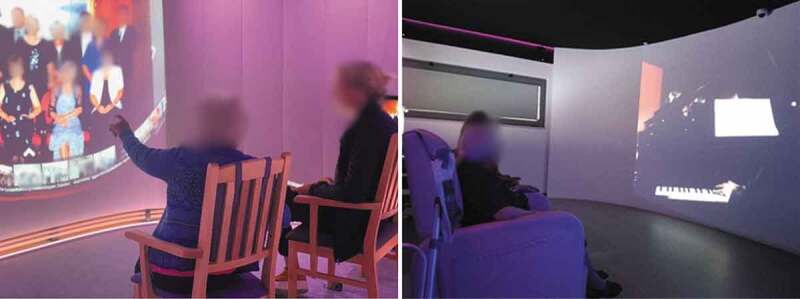
SENSE-GARDEN space in care home 1, Norway (left) and care home 2, Portugal (right)

Two care homes were involved in this study, located in Norway and Portugal. Care home 1 was a municipality-based care home for the elderly, located in a remote town on the west coast of Norway which has a population of less than 10,000 inhabitants. The facility provides residents with daily care, a communal dining area and a day centre where individuals can participate in leisure activities such as group singing. Care home 2 was a care facility belonging to a large, non-profit organization. The care facility is based in one of Portugal’s largest cities, with a population of over half a million people. The organization has over 20 care facilities in this city, and each facility operates according to a humanitarian goal through focusing on promoting resident quality of life. The SENSE-GARDEN space at each care home is shown in Figure 1.

### Participants

A total of 12 dyads were included in this interview study, with each dyad consisting of one person with dementia and a caregiver. The relationships between dyads in Norway were familial or spousal. The relationships between dyads in Portugal consisted of two familial relationships, three close friendships, and four professional caregiving relationships. Due to the unavailability of informal caregivers, four residents had only formal caregivers (care staff at the facility) accompanying them to the sessions.

The persons with dementia were recruited by managerial staff at the two care facilities if they were aged 55 or more and living with dementia in stage 2 (moderate) or stage 3 (severe) according to the Clinical Dementia Rating scale (CDR) (Hughes et al., 1982). Demographics of the participants are shown in Table I. Pseudonymisation was used to process personal data. The generated codes include two initial letters designating the country, a letter for type of participant (person with dementia or caregiver) and a sequential number. The mean age of the 12 participants with dementia was 84.1 years, and most had moderate dementia according to the CDR scale (N = 10).

**Table I.** Overview of participants

Dyad number	Care home	PWD participant code	Age	Type of dementia	CDR Level	SG Use (weeks)	Caregiver participant code	Relationship
1	1	NOp01	94	Unspecified	2	16	NOic01	Mother-daughter
2	1	NOp02	83	Unspecified	2	12	NOic02	Husband-wife
3	1	NOp03	79	Alzheimer’s Disease	2	12	NOic03	Father-daughter
4	2	PTp01	88	Dementia with Lewy Bodies and Parkinson’s	2	12	PTic01	Close family friends for a considerable amount of years
5	2	PTp03	71	Vascular Dementia	2	16	PTfc03	Care home staff
6	2	PTp04	89	Dementia with Parkinson’s	2	16	PTic04	Close friends
7	2	PTp05	81	Unspecified	2	16	PTfc05	Care home staff
8	2	PTp06	69	Alcohol-related dementia	3	12	PTic06	Close friends
9	2	PTp07	77	Unspecified	2	12	PTfc07	Care home staff
10	2	PTp08	92	Unspecified	2	16	PTic08	Father-Daughter
11	2	PTp09	97	Unspecified	2	16	PTic09	Aunt-niece
12	2	PTp10	89	Dementia with Parkinson’s	3	12	PTfc10	Care home staff

PWD: Person with dementia; ic: Informal caregiver (family/friend); fc: formal caregiver (professional care staff); NO: Norway; PT: Portugal; CDR: Clinical Dementia Rating Scale; SG: SENSE-GARDEN

### Intervention

**Figure 2.**
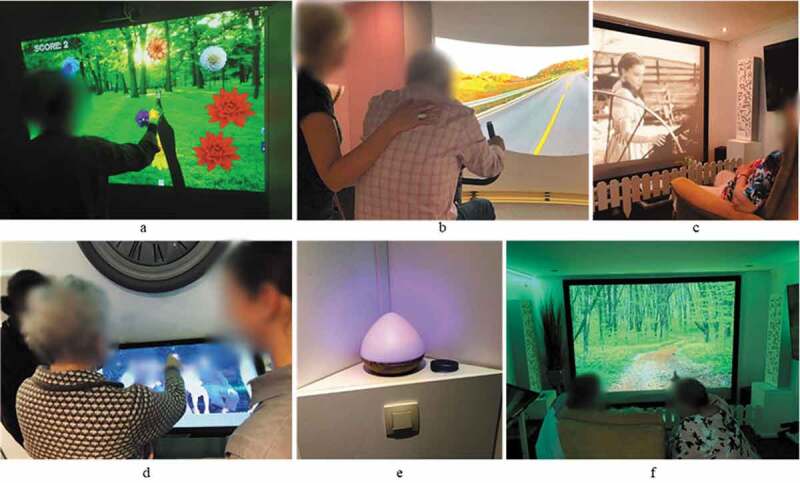
Activities within the SENSE-GARDEN: Move to improve (a); Life road (b); Films of my life (c); Memory lane (d); Scent to memories (e); Reality wall (f)

The SENSE-GARDEN intervention is a psychosocial intervention that provides individualized, meaningful activities to people with moderate to severe dementia within a multisensory environment (the SENSE-GARDEN space). The SENSE-GARDEN consists of numerous components and activities (shown in Figure 2) including an interactive game designed to improve balance and physical activity, a stationary bike placed in front of a film of a known place, old films, a touchscreen device with family photographs, a scent dispensary system which dispenses familiar scents, a large-screen projection of scenic imagery, and surround sound music and soundscapes.

The SENSE-GARDEN intervention is facilitated by a member of care staff at the care home (who is referred to as a formal caregiver). The formal caregiver encourages the person with dementia to interact with the various activities within the SENSE-GARDEN space. Family members (informal caregivers) may also join the sessions, engaging in the various activities together with the person with dementia and the formal caregiver.

The preparation and facilitation of SENSE-GARDEN sessions is undertaken by the formal caregiver. To ensure that the intervention is individualized to the person with dementia, the first step in preparing the SENSE-GARDEN sessions involves working with the family of the person, who are asked to provide information about the life story of the individual along with photographs and videos that could be used. The collated information and media are used to create a user profile for the person with dementia, designated as the “Arts of Life Memory Album” (ALMA). Formal caregivers involved in the project have reported that the process of creating a profile takes approximately an hour for each resident.

The next step in preparing the sessions involves using the contents of the ALMA to create media flows, which are sequences of photos, videos, and music that can be used for the activities carried out during the SENSE-GARDEN sessions. This is done using a tablet app developed by the SENSE-GARDEN technical team. Formal caregivers have reported that this process takes approximately 15 minutes for each media flow. This same app is also used in the facilitation of the sessions, with the formal caregiver using it to control the contents used for each session. Each session is intended to last between 30 and 60 minutes.

In addition, the caregiver is asked to use the app to register the feedback of the person with dementia, for example, how the individual responded to the media contents used in the session. This feedback is used to improve the selection of media contents for subsequent SENSE-GARDEN sessions. When planning future sessions, the app should automatically prioritize content that has been assigned positive feedback on the displayed list of available content, so that the formal caregiver can easily access the content that is most enjoyed by the resident. Depending on time availability, the wishes of the resident, and new knowledge gained about resident through the sessions, the formal caregiver can choose to create new sessions with updated media contents or use previously made media flows used in earlier sessions.

Prior to the study, care staff received written instructions and video tutorials on how to prepare and conduct sessions using the aforementioned SENSE-GARDEN app. During the study, the care staff used an online helpdesk to report any technical issues they encountered. These issues were addressed by the project’s technical team, who were able to offer support as needed.

### Data collection

A total of 16 interviews (12 individual interviews and 4 group interviews) were conducted with 7 residents with dementia and 13 caregivers across the two care facilities between December 2019 and May 2020. An overview of these interviews is shown in Table II.

**Table II.** Overview of interviews

Dyad	Interview	Interview type	Participants	Interviewer(s)
1	1	Individual	PwD	SG Facilitator
	2	Individual	ICG	SG Facilitator
2	3	Individual	PwD	SG Facilitator
	4	Individual	ICG	SG Facilitator
3	5	Individual	PwD	SG Facilitator
	6	Individual	ICG	SG Facilitator
4	7	Group	PwD, ICG	Researcher + SG Facilitator
5	8	Individual	PwD	Researcher + SG Facilitator
	9	Individual	FCG^a^	Researcher + SG Facilitator
6	10	Group	PwD, ICG	Researcher + SG Facilitator
7	11	Group*	FCG, FCG**	Researcher + SG Facilitator
8	12	Individual*	ICG	Researcher + SG Facilitator
9	13	Group	PwD, FCG	Researcher + SG Facilitator
10	14	Individual*	ICG	Researcher
11	15	Individual*	ICG	Researcher + SG Facilitator
12	16	Individual*	FCG^a^	Researcher

PwD: Person with dementia; ICG: Informal caregiver; FCG: Formal caregiver; SG Facilitator: SENSE-GARDEN Facilitator

^a^The caregiver did not join any SENSE-GARDEN sessions

*Interview had to be conducted over the phone due to the coronavirus pandemic. It was not possible to interview the PwD in the dyad during this time.

**The PwD in this case had become seriously ill at the end of the study and it was not possible to conduct an interview with him. Another member of care staff who had helped facilitate some SENSE-GARDEN sessions with the resident joined the interview.

Interviews were semi-structured and conducted in a conversational style (see Supplementary Material for Interview Guide). The interviews in Norway were conducted by a member of staff at the care home who joined the SENSE-GARDEN project in August 2019. This member of staff, who has a background in nursing, had been facilitating all of the SENSE-GARDEN sessions at the care home. The interviews in Portugal were conducted by two individuals who both joined the project before the start of the trials, which commenced in August 2019. The first is researcher and co-author LA, who has a background in sociology. The second is a psychologist recruited for the SENSE-GARDEN project. She had been facilitating most of the SENSE-GARDEN sessions in Portugal, along with members of staff at the care home.

Where possible, both the person with dementia and the informal caregiver were interviewed. In Norway, all interviews were conducted on an individual basis- one with the person with dementia, and the other with the informal caregiver. In Portugal, a mix of individual and group interviews were used. Additionally, two formal caregivers in Portugal did not join for SENSE-GARDEN sessions but were still interviewed. In these two interviews, the guide was adapted to ask questions about their perceptions of SENSE-GARDEN in general, and what effects—if any—they had noticed on the person with dementia.

After the onset of the coronavirus pandemic, it was no longer possible to conduct interviews in-person. There were a remainder of 5 dyads to be interviewed, and we were able to reach the caregivers in each dyad by phone to conduct telephone interviews. However, it was not possible to interview the person with dementia in the dyad.

All interviews, with the exception of one, were audio recorded, transcribed, and then translated into English for analysis. The one interview that was not recorded was a telephone interview with an informal caregiver (PTic08), who requested that the conversation was not recorded. In this instance, the interviewer took note of the participant’s answers.

### Ethics and consent

Each test site followed ethical guidelines in accordance with their national regulations. In Norway, the study was approved by the Regional Committee for Medical and Health Research Ethics (REK nord reference 10015). Ethical approval from a formal ethics review committee was not required for this kind of intervention study in Portugal. However, the study followed the principles of the Declaration of Helsinki.

Written informed consent was given by the participants. If the participant lacked capacity to consent, consent was gained through proxy. The current study adhered to national regulations concerning consent to research. Norway’s Health Research Act (Lovdata, 2008) states that in the case that a person does not have the capacity to provide consent, the person’s next-of-kin shall have authority to grant consent. The act also states that people who lack the capacity to give consent may only be included in research if a) the potential risks or disadvantages are insignificant; b) the individual involved is not averse to it; and c) there is reason to assume that the results of the research may be of use to the person concerned or other people with the same disorder or disease. Similarly, Portugal’s legislation concerning clinical trials (“Aprova a Lei da Investigação Clínica,” 2014) states that if a person is incapable of providing consent, consent must be provided by the person’s legal guardian. Legislation also states that a person without capacity to provide consent may only participate in the study if the intervention is designed to prevent the disease, to provide rehabilitation, and to prevent any foreseeable risk related to the disease, as well as the degree of suffering caused by the disease. Given the SENSE-GARDEN’s aim to improve the well-being of people with moderate/advanced dementia, it was considered ethically sound to conduct the study with people who may not have the capacity to provide consent. In both sites, the guardian or legal representative was the informal caregiver, with whom the participant with dementia was close to prior to the study. Thus, it was expected that the informal caregiver would have decided whether or not to agree to the study based on the interests of the person with dementia.

Despite informed consent being provided by proxy, the participants with dementia could still refuse to participate. Before each SENSE-GARDEN session, the professional facilitating the session would approach the resident, greet them, and ask if the participant would like to join them to the SENSE-GARDEN to take a look at some photos and play some music. The professional caregiver would then decide whether or not to take the resident to the SENSE-GARDEN, based on the resident’s response and behaviour. This could be considered in line with Dewing’s (2007) guidelines for ongoing consent monitoring, in which ensuring initial consent is revisited and re-established on every occasion throughout the study. Additionally, the sessions could be stopped at any time. If the participants showed any sign of distress or discomfort during the session, the session would be immediately stopped. To ensure the well-being of participants, all SENSE-GARDEN sessions were facilitated by care professionals with experience of working with people with dementia. In Norway, sessions were conducted by a nurse who has 14 years of experience caring for people with dementia. In Portugal, sessions were conducted by two psychologists who have 8 and 4 years of experience in dementia care, respectively, and an occupational therapist who has 17 years of experience.

The interviews were conducted by the professionals who had been facilitating the SENSE-GARDEN sessions, as to provide the participants a sense of familiarity during the interview. Professionals received an interview guide from the first author of the study, as well as advice on how to conduct the interview. The interviewers were also able to contact the first author if they needed further help with the interviews. No interviews were conducted with residents with dementia during the COVID-19 pandemic.

### Analysis

Reflexive thematic analysis (Braun & Clarke, 2006, 2019; Braun et al., 2019) was used to analyse the interview transcripts. The aim of reflective thematic analysis (RTA) is to generate themes that reflect a pattern of shared meaning around a central organizing concept. In RTA, researcher subjectivity and reflexivity are used as resources (Braun et al., 2019). The following six steps (as outlined by Braun & Clarke, 2006, 2019) were taken:

## 1. Familiarization with data

The first author compiled the transcripts from the two sites into NVivo 12 (QSR International, 1999). In order to get a sense of the data, the transcripts were read repeatedly, and initial ideas and reflections were noted down.

## 2. Generating codes

Reflexive thematic analysis allows for varying approaches to coding. In the present study, Fereday and Muir-Cochrane (2006) hybrid approach of deductive and inductive coding was used. This approach integrates theory-driven (deductive) codes with data-driven (inductive) codes. In this case, Deweyan philosophy and symbolic interactionism were used to inform the development of the codebook for deductive coding. This was done by using the key principles and ideas behind transactionalism and symbolic interactionism to develop codes a priori that would be relevant to the research questions and the context of the SENSE-GARDEN intervention. Table III demonstrates the development of the theory-driven codes, giving the theoretical foundation and definition for each code.

**Table III.** Development of deductive codes

Code name	Theoretical foundation for code	Code definition
Temporal focus	Building on the work of Mead, symbolic interactionists believe that the past is symbolically reconstructed in the present, and assigned new meaning based on an anticipated future (Mead, 1932; Maines, 2001). Given the SENSE-GARDEN’s focus on the life story of the person with dementia, it is important to understand how the participants refer to past, present, and future as a result of interacting with personally significant media.	Referring to past, present, and/or future
Shared identity	Symbolic interactionists believe that meaning, emotions, and pasts can be shared between individuals through joint interaction (Mattley, 2002). As such, social—or shared—identities can be co-constructed as a result of these interactions and shared values. The code “shared identity” is to reflect on how dyads in the study—particularly familial dyads—may feel that their identity is shared based on the meaning they assign to their experiences.	Referring to identity as co-constructed between two or more people
Meaning	People assign meanings to objects, places, events, others etc. and these meanings are constantly reinterpreted as a result of interaction with these objects etc. (Blumer, 1986). The meaning that an individual has attributed to the world around them may influence how they experience the SENSE-GARDEN intervention.	Attributing meaning to media contents, object, place, event, or memory
Interpersonal relationships	Given that symbolic interactionism concerns how behaviour is shaped through interaction with others, the exploration of how participants perceive and describe their relationships with others may provide insight into how these relationships are experienced in the context of SENSE-GARDEN.	Referring to relationships with other individuals
Behaviour and actions	Symbolic interactionism concerns human behaviour and how it is shaped through social interaction. The way that participants perceive and interpret their own behaviours and the behaviours of others, as well as how they interpret their interactions, will contribute to the overall understanding of experiences within SENSE-GARDEN.	Referring to verbal and/or non-verbal behaviours and actions
Space and aesthetics	Transactionalism emphasizes that human experience is shaped through an individual’s interaction with their environment (Dewey, 1934). Understanding the participants’ awareness and perceptions of their surrounding environment is therefore vital to making sense of their experiences both in and outside of SENSE-GARDEN.	Referring to SENSE-GARDEN space or space of other environments
Emotions	Both Dewey and Mead viewed emotion as embedded in social interaction (Ward & Throop, 1989). According to a symbolic interactionist perspective, emotions are not only experienced and reflected upon in response to situations, but the ways in which they are expressed—or not expressed—can shape social interactions and relationships (Mattley, 2002). Exploring how the participants experience and make sense of their emotions, as well as the emotions of others, may provide insight into the relationships they hold with one another.	Referring to both positive and/or negative emotions and feelings

Three coders (GG, LA, JAS) independently read the transcripts and performed deductive coding using the initial codebook of theory-driven codes. The use of multiple coders in RTA is to develop a more nuanced understanding of the data through collaboration (Braun & Clarke, 2019). Additionally, qualitative analysis can be enhanced by including multiple coders with varying backgrounds (Berends & Johnston, 2005). The coders in this study have a background in music psychology (coder 1), sociology (coder 2), and care and assistive technologies (coder 3). Once coding was complete, the coded transcripts were shared amongst the coders, who then discussed their impressions of the data, as well as their suggestions for inductive codes, based on the data.

As a result of discussion, two deductive codes (temporal focus and shared identity) were removed from the codebook. This was based on the fact that they were seldom used in the coding amongst the three coders and, after discussion, the coders felt they did not accurately represent the participants’ views and experiences of SENSE-GARDEN. As Braun and Clarke (2019) state, reflexive thematic analysis should be a flexible process that values the importance of deep reflection on, and engagement with, the data. Therefore, in order to be true to the dataset, the decision was made to remove the codes.

**Figure 3.**
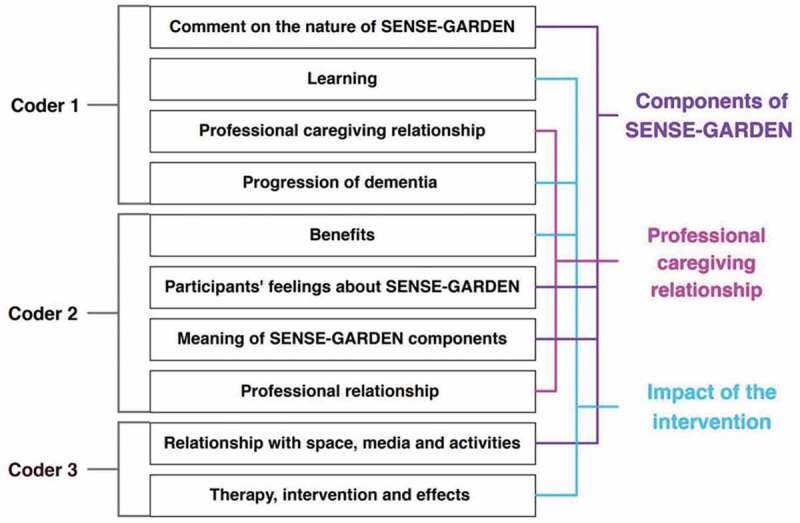
Generating inductive codes

The inductive codes suggested by each coder were merged to form three inductive codes (see Figure 3). As a result of discussion, two deductive codes (temporal focus and shared identity) were removed from the codebook. A final version of the codebook is shown in Table IV. The entire dataset was once again coded in NVivo according to this new version of the codebook. This was conducted by GG.

**Table IV.** Final codebook

Code name	Definition	Description	Example
Meaning	Meaning is attributed to a place, event, media, or memory	The participant talks about the meaning/significance of media (music, photographs etc.), places (e.g., hometown), events or memories	“I know that Fátima* is very important to her, and the religious part touches her a lot”
Interpersonalrelationships	Discusses interpersonal relationships with other individuals	Interpersonal relationships with other individuals (living or deceased, inside and/or outside of SENSE-GARDEN). The emotional and/or social nature of the relationship may be discussed	“I’ve learned more about appreciating our 60 years of life and all of the 21,000 days we have had. Most of them have been happy. It has not been said that we have never quarrelled, but we have never gone to bed as enemies. We have taught ourselves to pay attention to each other”
Behaviour and Actions	Interaction (verbal and/or non-verbal)	May refer to gestures, body language, facial expressions as well as verbal communication. Can refer to interaction either inside or outside of SENSE-GARDEN	I generally think it has become easier to talk to her even when she is not in the SENSE-GARDEN. She is more sharp and able to hold the thread of the conversation better than she did before.
Space and aesthetics	Participant discusses space and/or comments on aesthetics	Can refer to the SENSE-GARDEN space, or space of other environments (e.g., other areas of the care facility)	“It’s the design of the room, the fact that there are no sharp edges, no corners, it’s carpeted. It is shielded from the rest of the world. One goes into something else, one forgets time.”
Emotions	Emotions are discussed	Emotions experienced either inside or outside SENSE-GARDEN are discussed. The nature of the emotion can be mixed (does not have to be only positive or negative).	“I even cried while playing the children’s song. it was a powerful experience … it was strong for me when my mother sang along to these songs. I think my mom is happy when she is here, happy and bright at heart.”
Professional caregiving relationship	Discusses the care given to the PwD by the professional caregiver	Refers to how professional caregiver interacts with the PwD, how they facilitate the SENSE-GARDEN session or the caregiving relationship outside of sessions	“I do not believe all the caregivers have become involved in his life situation and there is always a reason why they are angry or sad. I think the staff misinterprets the user. One must find the reason why the user is the way he is.”
Impact of the intervention	Discusses benefits or issues as a result of the SENSE-GARDEN intervention	Refers to either immediate or long-term effects (both positive and negative) of the intervention on the person with dementia and/or caregivers	“I generally think it has become easier to talk to her even when she is not in the sensory garden. she is more sharp and able to hold the thread of the conversation better than she did before. She doesn’t ask the same question again. if I switch topic and then comes back to the previous conversation the topic, she manages to remember what we talk about 3 minutes ago. It has become much easier to talk to her now on the phone. It is probably the change that I think I have seen.”
Components of SENSE-GARDEN	Discusses aspects of the SENSE-GARDEN	Refers to activities, media and/or technology within the SENSE-GARDEN space	“It was especially the pictures combined with the music I liked the best. The family pictures I liked a lot. It is so wonderful, and it is accurate that I want to burst with enthusiasm. Quite phenomenal.”

* Fátima is a Portuguese town that’s home to the “Sanctuary of Fátima”, a well-known Catholic pilgrimage destination.

PwD: Person with dementia

## 3. Generating initial themes

Initial themes were generated by GG, who used that to identify patterns across the dataset. She collated the codes, along with the coded excerpts of data, into potential themes through careful reflection on the dataset and the research questions. Braun and Clarke (2006) note the importance of this phase of analysis being conducted at the broader level of themes, in which codes may be discarded, combined to create themes, or become themes of their own. As such, themes and subthemes were identified across different codes. For example, excerpts of data coded for “Emotions”, “Behaviour and Actions”, or “Interpersonal Relationships” were interconnected by the prevalence of communication, which later became a subtheme under the theme “Openness”.

## 4. Reviewing themes

The reviewing of themes took place through a joint discussion amongst authors. GG consulted with the other two coders to ensure that these themes reflected the dataset as a whole, as well as being representative of the participants on an individual level. A thematic map was made to aid the process of reviewing themes, as well as to gain insight into how the themes interlink with one another and form an overall narrative about the data.

## 5. Defining and naming themes

This process involves refining the specifics of each theme and the overall story the analysis tells. This was again done in a collaborative manner between co-authors.

## 6. Producing the report

The report was produced primarily by GG. The aim was to provide the reader with a sense of the story about the data that was generated by the authors, using direct quotes from the participants to support the portrayal of this story. The final report was approved by all co-authors.

### Results

**Figure 4.**
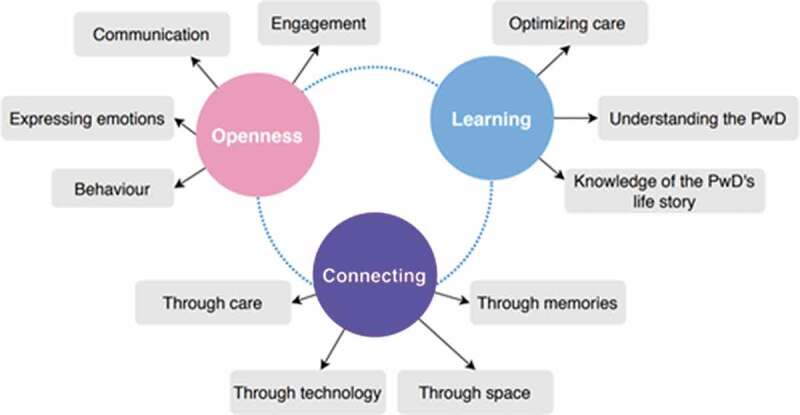
Thematic map of themes generated through reflexive thematic analysis

Three themes were generated from reflexive thematic analysis: openness, learning, and connecting. An overview of these themes and their respective subthemes is shown in Figure 4. The first theme, “openness”, reflects the way in which participants felt encouraged to be more open with one another while using SENSE-GARDEN. The second theme, “learning”, reflects the way in which caregivers felt that their knowledge of the person with dementia had improved through the use of SENSE-GARDEN and thus wanted to apply similar techniques to optimize the care environment in general. The third theme, “connecting”, captures the various ways in which participants felt connected to one another while using the SENSE-GARDEN. The thematic map also illustrates the interactive and interdependent nature between the three themes. For instance, in order to facilitate openness, there must be an opportunity for the resident and the caregiver to connect with one another. However, this connection will be hindered if the caregiver has limited knowledge on the person with dementia. As such, learning is required. In order for learning to take place, the caregiver should have an open attitude towards the person with dementia, encouraging them to be expressive and engaged.

### Openness

The theme “openness” reports on the participants’ overall belief that the SENSE-GARDEN encourages those who use it to become more open with one another. This theme has four subthemes: 1) “communication” reporting on how SENSE-GARDEN provided benefits in terms of communicative abilities, 2) “engagement” reporting on how participants actively engaged with the activities and media contents within the SENSE-GARDEN, 3) “expressing emotions” relating to how SENSE-GARDEN not only evoked emotional reactions but how these emotions were expressed and interpreted by participants, and 4) “behaviour” relating to how caregivers noticed a change in behaviour amongst the residents during and after using SENSE-GARDEN.

### Communication

Both people with dementia and caregivers spoke about openness in terms of communication, particularly with being able to talk freely. One gentleman with dementia, who used to be a teacher, was adamant in his opinion that the SENSE-GARDEN encouraged people to talk more openly:

“It was an open forum, you could talk about everything. It must be like that you know. It must be so that one can open up a bit, then you get people talking too. There was openness to talk about everything, and that was definitely the meaning I guess … I think it’s really good that people get to talk a little, and then you get it out.” NOp02

As well as opening up inside the SENSE-GARDEN, benefits in communication were also observed outside of the SENSE-GARDEN sessions:

“I generally think it has become easier to talk to her even when she is not in the SENSE-GARDEN. She is more sharp and able to hold the thread of the conversation better than she did before. She doesn’t ask the same question again. If I switch topic and then come back to the previous conversation, she manages to remember what we talked about 3 minutes ago.” NOic01

The SENSE-GARDEN itself was also talked about outside of the sessions, facilitating communication between care home residents. For example, the wife of one participant commented on how her husband would tell the other residents in the care home about his visits to the SENSE-GARDEN:

“He (NOp02) … was interested in telling the other residents about his stay in the sensory room and he also liked to tell the family. He was shining like the sun.” NOic02

The lady also commented that her husband “was shining like the sun”, suggesting that not only was he talking about SENSE-GARDEN but he was doing so in a way that conveyed his enjoyment of being inside the space.

### Engagement

Caregivers noticed that the participants with dementia were willing to participate in the SENSE-GARDEN sessions and engage with the activities inside the space. As such, they became more open through this engagement:

“Yes, he participated and … showed a lot of interest … Then it wasn’t so isolated, I mean, in his little world … he is more open.” PTfc03

Even in the case where memory was notably impaired, one caregiver noticed that the person with dementia still managed to engage with the music despite having a suggested lack of self-awareness. The quote below suggests a symbiosis between past and present, with the person with dementia engaging in the present moment (i.e., singing along to the music) whilst simultaneously returning to the past:

“I realized that even though [PTp06] didn’t know who she was, deep down, she felt the music, she felt good, as if she remembered that place. And then I also saw that when she started singing … There was a very interesting interaction … She sang and it seemed that … she went back to the past.” PTic06

Participants with dementia also expressed their desire to engage with the stimuli inside SENSE-GARDEN, with music being particularly popular:

“Without the music everything would have been boring. I think it was very important. There is something going on inside, one knows it in the whole body. I want to sing and dance.” NOp01

### Expressing Emotions

From the transcripts, it was clear that SENSE-GARDEN had an effect on the emotional state of participants. Accounts of various emotional experiences encountered within the SENSE-GARDEN were prominent throughout the interviews. The caregivers offered thoughtful and reflective interpretations of how the participants with dementia experienced the intervention:

“I thought she was much more open … it’s not just the memory, it’s an opening to this part of feeling that she closes to protect herself.” PTic01

The above quote illustrates a sense of openness in terms of experiencing and expressing feelings. It also highlights how emotions are not only connected to memories but to the self as a whole, beyond that of the ability to recall or recollect information.

Participants with dementia also expressed positive emotions experienced within the SENSE-GARDEN:

“It was especially the pictures combined with the music I liked the best. The family pictures I liked a lot. It is so wonderful and it is accurate that I want to burst with enthusiasm. quite phenomenal.” NOp01

Again, the “bursting” with enthusiasm resonates with the theme of openness in a particularly strong manner. The caregivers also experienced strong emotional experiences, particularly in reaction to seeing the person with dementia sing. Their interpretation of this gesture was loaded with meaning—the action of singing reminded the caregivers that their family members could still engage in the present moment:

“I even cried while playing the children’s song. It was a powerful experience. There were several songs we sang when I was little. it was strong for me when my mother sang along to these songs.” NOic01

“I had my throat many times [wanting to cry] … Because she remembered, because she sang.” PTic01

Additionally, the above quotes illustrate the complex nature of emotions, that is, being happy but wanting to cry. This mixture of emotions was also associated with the media contents shown inside the sessions. The participants often spoke about pictures and videos of the past. The association with these pictures had a new meaning when being recollected in the present, as they served as a reminder of a time before dementia had made an impact on their lives:

“You had a lot of nice pictures. It is a bit strange and sore to see pictures from when the kids were small. It was the time when everything was fine and good and safe. You put the kids to bed in the evening and they were happy and fell asleep well. I thought the time I had then would always be with us.” NOic02

“It is positive because it brings up a lot of memories that he has really forgotten or displaced. He had so much inside as that he has closed inside him, something that the SENSE-GARDEN now has opened. Both with joy and some sorrow.” NOic03

One participant with dementia stated that he became emotional when watching a video of a ferry trip to Nordkapp—somewhere he had been numerous times in his life. In sharing his experience of watching the video, it is clear to see that he held strong emotional attachment to this memory, and he felt comfortable in being able to express his feelings both in the session and again in the interview:

“Fabulous, the first trip was by ferry and up to Nordkapp. I remember that trip very well, I became emotional and cried a lot.” NOp03

In some instances, sessions included the use of photographs containing family members who had passed away. One woman expressed that she felt sad as a result of seeing her father upset by nostalgia attached to the pictures:

“I get sensitive when being in the SENSE-GARDEN. I felt joy and a bit of sadness. I got sad when I saw what grief [NOp03] is carrying. He remembers more when he looked at pictures, fond memories of the family he lost.” NOic03

The above quotes illustrate that whilst the SENSE-GARDEN is successful in provoking memories, it also provokes a sense of nostalgia attached to these memories. As such, a mixture of emotions is experienced. The quotes also emphasize the social nature of emotions—how they can be shared, interpreted, and expressed in relation to others. The openness inside the SENSE-GARDEN seems to prompt a level of vulnerability amongst participants, which allows for a free expression of emotions, without fear of judgement.

### Behaviour

Openness was also experienced with regard to verbal and non-verbal behaviours. Caregivers noticed positive changes in the behaviour of participants with dementia after attending the SENSE-GARDEN sessions. One informal caregiver felt that her friend with dementia had returned to how she used to be 15 years ago as a result of using SENSE-GARDEN:

“It’s like night and day, it’s the [PTp01] of old times, 15 years ago. There were activities that she … in these years, in recent times, in recent years that she had never done again and now she did.” PTic01

A member of care staff commented on how a resident with dementia became less aggressive after the SENSE-GARDEN sessions, indicating the possibility for the staff member to interact with the resident in a different way:

“She used to spend more time gesturing, more aggressive, talking aggressively and now she is not, she is calmer, she is different.” PTfc07

However, when the intervention period ended and the visits to SENSE-GARDEN had stopped, caregivers noticed a negative change in behaviour. Caregivers commented that residents were disappointment when the intervention came to an end, and that they wanted to continue sessions. For example, the wife of one participant stated:

“He looks blank. He had the SENSE-GARDEN to look forward to. I notice that he falls into himself … he becomes more confined when he does not receive stimuli. When I ask him how he is doing, he blames his back. But I think he blames his back when he doesn’t feel so good.” NOic02

The notion of “he falls into himself” indicates a kind of closing, opposite to the openness which has been apparent throughout this theme. This indicates the importance of maintaining individualized activities in dementia care in helping residents to maintain positive behaviours that can help them connect with others.

### Learning

The theme “learning” refers to the ways in which visits to SENSE-GARDEN improved knowledge on the person with dementia in terms of knowing their life story, and also in terms of understanding them in everyday interactions. This theme has three subthemes: 1) “optimizing care” reporting on how caregivers believed that SENSE-GARDEN, or similar activities offered in the intervention, should be incorporated into regular care within the care home, 2) “understanding the person with dementia” reporting on how spending one-on-one time with the person with dementia improved not just the caregivers’ biographical knowledge of the resident, but also their understanding of the person’s behaviour, and 3) “knowledge of the person with dementia’s life story” relating to how using the SENSE-GARDEN can shed more light on the life story of the resident for both family caregivers and professional caregivers.

### Optimizing care

Insights from SENSE-GARDEN experience led caregivers—both in Norway and Portugal—to reflect on how the usual care environment, beyond the project intervention, could be improved. Participants felt that the residents’ living environment should incorporate personalized items such as pictures and movies, in order to promote their well-being. For example, the daughter of a participant commented:

“The walls of the care home should have been wallpapered by pictures. Now I have made photo albums for her, but I think the best thing is to have it on the wall. A good thing would be to have photos on the tv in the care home. I think the older ones would like that.” NOic01

One lady noted how the care environment itself was a contributing factor to the progression of her husband’s dementia:

“His illness is aggravated by sitting in a care home. He makes bad thoughts after moving into the nursing home.” NOic02

Others felt that SENSE-GARDEN should be integrated into all care homes, specifying that a care home should be a place in which residents should be valued:

“I really liked it, I think it was very good, very positive, I think this work is worthwhile, should be put in every residence. For me, I think that residences are not a place where people are there … handing them over … it is not a warehouse. At the end of people’s lives, people have to have dignity, be happy and die well. Be valued.” PTic04

These quotes highlight a visible need for enhancing the quality of the care environment in both settings.

### Understanding the person with dementia

During the interviews, there were criticisms from family caregivers towards the care that their loved one receives. One wife of a participant felt that her husband was given the opportunity to engage with activities and gain a sense of achievement in SENSE-GARDEN, which is something he does not have an opportunity to do in normal care:

“He does not have the same opportunity in the nursing home, that he can master something [as he does in SENSE-GARDEN]. No one expects anything from him.” NOic02

The lady also says no one expects anything from her husband, which resonates with the misperception of people with dementia as being passive sufferers of the disease. However, in SENSE-GARDEN, the formal caregiver empowered her husband, as he expressed during his interview: “I was encouraged to tell” (NOp02). Similarly, the daughter of another participant felt that the staff at the care home did not take the time to understand her father:

“He had so much … closed inside him, something that the SENSE-GARDEN now has opened … It doesn’t seem like everyone understands it. I do not believe all the caregivers have become involved in his life situation and there is always a reason why he is angry or sad. I think the staff misinterprets [NOp03]. One must find the reason why he is the way he is.” NOic03

In contrast, caregivers felt that spending time with the person with dementia inside the SENSE-GARDEN led to an improved understanding of the individual. As such, this provided benefits for both individuals in the caregiving relationship:

“I started to know [PTp04] better … Sometimes, we don’t understand why there are certain reactions … . I think it was very important for us to get to know each other better, I understand what [PTp04] likes most, how [PTp04] works in terms of connections with people. [PTp04] accepts me better now than she accepted me before … When I know that [PTp04] smiles I am happy and that is true. When I know that [PTp04] is bored and sad, I also wonder what can I do, what is going on? … I think that [PTp04] also felt more confident about telling me things, therefore, a greater opening. I think it was positive for both sides.” PTic04

Looking at these results from a symbolic interactionist perspective emphasizes the importance of social interaction in the maintenance of identity and relationships. By learning to interact with the resident through the use of activities that provide meaning to the resident’s everyday life—as opposed to only providing basic care—staff may understand the person with dementia in a way that provides benefits to the caregiving relationship.

### Knowledge of the person with dementia’s life story

The technology inside of SENSE-GARDEN provides the opportunity to interact with the life story of the person with dementia in a readily accessible and sustainable manner. Engaging with the media contents based on the life story of the person with dementia provided the opportunity for the caregiver to get to know the individual better. A touching account from the wife of a participant with dementia suggests that the SENSE-GARDEN can provide new knowledge on the person with dementia, even in spousal relationships:

“The experience itself has probably caused me to open my eyes to small things that I have not noticed before. Things I had no idea meant anything to him, with us having gone further into ourselves. And I learned more about appreciating our 60 years of life and all of the 21,000 days we have had. Most of them have been happy. It has not been said that we have never quarreled, but we never went to bed as enemies. We have taught us to pay attention to each other.” NOic02

This new knowledge is particularly important for professional caregivers, who may not know as much about the resident compared to a close friend or family member. One member of staff mentioned she had done some research on topics she knew were of interest to a resident, and found that this prompted the resident to share more of his life story with her as the sessions went on:

“As the sessions went by, he added information … he was talking about the picnics that he had with the wife, with the children, with the mother-in-law … And I think this middle part [of the sessions] was more significant than the initial part.” Formal caregiver who facilitated sessions with PTp05

The caregiver commented that later sessions were more significant compared to the initial ones, and this could be due to the increased amount of knowledge gained on the life story of the resident. However, whilst the SENSE-GARDEN can help staff engage with the life story of the person with dementia, it is important to acknowledge the amount of time and effort it takes to collect media and prepare sessions. One staff member mentioned the difficulty of planning sessions:

“… about the preparation of the sessions, it is difficult to have a planned drawing [organization of the sessions], for example, for 30 sessions. The meaning of this [SENSE-GARDEN intervention] is to always be changed, created.” PTfc05

### Connecting

The theme “connecting” encapsulates how connections are made between individuals through using SENSE-GARDEN together. This theme has four subthemes: 1) “through care” reporting on how the formal caregivers facilitated sessions in a way that enhanced the caregiver-resident relationship and the overall SENSE-GARDEN experience, 2) “through technology” reporting on how the technology used in SENSE-GARDEN prompted conversation and connected participants to their own sense of identity, 3) “through space” reporting on how participants considered the SENSE-GARDEN space as one in which they felt safe and connected, and 4) “through memories” reporting on how participants connected through talking about memories that were triggered and shared during the sessions, and how these memories remained intact after the sessions.

### Through care

The informal caregivers perceived the SENSE-GARDEN as a positive experience partly due to the way in which sessions were facilitated by the formal caregivers. Informal caregivers in both Norway and Portugal commented on the facilitation style, which was perceived as comforting, safe, and respectful. The informal caregivers also felt the care provided by the formal caregivers was a factor in the residents wanting to return to the SENSE-GARDEN for subsequent sessions:

“Another thing I have been thinking about is that you, [formal caregiver], have a comfortable attitude, you make my mother feel safe and respected. Not everyone is as good at meeting people as openly as you do.” NOic01

“The person he was waiting to see was [the formal caregiver], because he knew that during that time she was going to be with him and that she was going to be doing something that gave him pleasure, that he liked.” PTfc05

One niece of a resident with dementia commented on how the facilitation style from the formal caregiver resulted in the SENSE-GARDEN session feeling like a family gathering:

“Also, the way [the formal caregiver] conducted the approaches and the conversation, I think it was all very natural, it seems that we were a family there. (Laughs). [The formal caregiver] already knew some stories, things from other sessions … I think we were a family, that we were there watching a family album.” PTic09

These quotes indicate that meaningful care staff-resident interactions can be fostered inside the SENSE-GARDEN, which can then influence the caregiving relationship outside the SENSE-GARDEN sessions.

### Through technology

The digital technologies and media contents used in the SENSE-GARDEN sessions were thought to facilitate connection and communication between the participants. Even in the case where memory was impaired, the contents of SENSE-GARDEN provided conversation topics and aided the flow of conversation. One participant with dementia also spoke about how he was encouraged to share his life story when being inside the SENSE-GARDEN:

“The SENSE-GARDEN is great for getting people to tell and say things. And that is important … then things come out more. I was encouraged to tell.” NOp02

Additionally, a sense of connection was identified not only between individuals but also to a sense of self amongst the participants with dementia:

“I think she sees things here that calm her heart … They are memories. It’s her story.” PTfc07

The technology was used as a way of portraying the life story of the residents back to the participants, and as such, it was something that they were able to connect to. One man with Alzheimer’s disease expressed that he felt a lot of happiness as a result of recognizing himself in the media contents:

*Interviewer* How did SENSE-GARDEN make you feel?

*NOp03* A lot of happiness.

*Interviewer* What was it about SENSE-GARDEN that made you feel that way?

*NOp03* It was the films that I recognized me in.”

The above quotes suggest that the use of digital technologies to convey personalized media contents can be useful in promoting a sense of self, even in the moderate stage of dementia.

### Through space

Overall, the participants were positive towards the physical aspects and aesthetics of the SENSE-GARDEN room. They also spoke about the ways in which the space harnessed an energy in which they could connect with others:

“It is the energy inside the sensory garden, good energy. One feels safe, very safe frames. It has to do with light, and the colors and people in it.” NOic03

Others spoke about how they felt transported inside the space:

“It may well be that it is quiet, the colors have a lot to say. It is often the music and the light that comes into play. It’s the design of the room, the fact that there are no sharp edges. No corners, it’s carpeted. It is shielded from the rest of the world. One goes into something else, one forgets time.” NOic01

“Those forests that [facilitator] showed us and we were running. (Laughs) In the middle of that forest, wasn’t it? With that running water, a spring. All of this transports us to our imagination, our childish part. I’m very romantic (Laughs) Here it makes me dream, this space …” PTic04

This quote illustrates how connection is made to not only other individuals in the room but also to the part of one’s self that is perhaps not connected to so often, that is, the “childish” part. Additionally, the caregivers spoke of the SENSE-GARDEN space being a part of the person with dementia:

“It’s his moment, his space.” PTfc05

“I felt that she was in her space, that she felt that space as if it were hers, it was of her …” PTic06

These remarks resonate strongly with Dewey’s notion of “human-as-organism-in-environment”, in which an individual is fundamentally at one with their surrounding (Dewey, 1929). Furthermore, the quotes reflect a metaphysical understanding of space, one which goes beyond physical features.

### Through memories

Whilst memories were often triggered by the digital media contents shown in the SENSE-GARDEN, the participants expressed the significance of the memories themselves. The participants’ remarks resonated with symbolic interactionist perspective that memories—and the emotions and meanings attached to these memories—can be shared through social interaction. For example, one caregiver reflected on how she felt that “people are made” through the joint recollection and conservation of memories:

“That’s [sharing memories are] how people are made. I like to talk about things I’ve experienced together. The pictures are a trigger of the memory and conversation. I think if I have been on a holiday trip, it is nice to look at the pictures with the family and talk about them later.” NOic01

Again, benefits of the intervention were seen beyond the SENSE-GARDEN room. Memories that were triggered in the SENSE-GARDEN appeared to be lasting beyond the session and were able to help communication between caregivers and residents:

“When I was talking about a cousin of his, I forgot what he was called. Suddenly my husband remembered his name.” NOic02

Similarly, the niece of a participant noticed that her aunt was able to remember aspects of what they spoke about during the sessions. In this case, the niece had expressed her concerns over her aunt being potentially upset by bringing up memories of the family, which had experienced problems in the past. However, her concerns were eased when she noticed her aunt “was fine”:

“Afterwards, when we finished the session sometimes I spent a little bit of time with her, or in the other days that I would go there and then I would talk to her a little bit about [their family]. I brought up the subject and noticed that she was fine … I noticed she remembered things well and spoke well, it was neither pity nor nostalgia. She spoke as if it had been a fact of her life and that was it.” PTic09

Another caregiver explained how her close family-friend became more connected to her family through the remembrance of family members and songs:

“I have the notion that she began to give and gave much more appreciation to this Christmas … because she remembered and spoke to my brothers, my husband and my cousin … so the people who went there … she remembered the names of those people and their loved ones, and they promised to come and see her now.” PTic01

Additionally, it is not only the memories of the person with dementia that provides connection, but it is also the ways in which other individuals consider the person that have an impact. In a rather touching remark, one lady with dementia commented on how she liked to be remembered by others:

“I also liked the photos and to be remembered here in this house” PTp04

This quote illustrates the important role of others in constructing narrative identity amongst people with dementia. For this participant, the role of others in “remembering” her was important to her. Through being remembered by others, and through the sharing of photos and stories, this sense of narrative identity can be sustained even when the person progresses into more moderate and advanced stages of dementia.

## Discussion

Overall, the findings suggest that an individualized technological intervention such as SENSE-GARDEN has a promising impact on facilitating meaningful activities in dementia care, particularly with regard to stimulating emotional experiences, preserving a sense of narrative identity, and improving interpersonal relationships—both on a familial and professional level. The findings are consistent with previous studies that implemented meaningful activities tailored to people with dementia in care homes which found that staff are encouraged to see the unique personhood of the individual (Broome et al., 2017: Figueiredo et al., 2013; Fritsch et al., 2009; Helgesen et al., 2020; Kuosa et al., 2015). This can improve the caregiving interaction, resulting in benefits for both staff and resident (Figueiredo et al., 2013; Helgesen et al., 2020).

This study also holds relevance to recent calls for the study of technology use in dementia care. For instance, a fairly recent Lancet commission on dementia prevention, intervention and care called for the use of technology in helping to improve care delivery (Livingston et al., 2017). Similarly, the World Dementia Council (2018) called for exploration into how new technology can be used as a means of connecting with others. The findings from this study indicate the potential of using a new technology combined with multisensory stimuli, such as SENSE-GARDEN, to provide a way for caregivers to connect with people with dementia. To gain insights into how this connection takes place, the results are discussed in relation to symbolic interactionism and Deweyan concept of transactional relationships.

### Symbolic interactions within SENSE-GARDEN

The findings suggest that the SENSE-GARDEN intervention is loaded with meanings constructed through the use of media contents to provide multisensory stimuli, through emotional reactions during the sessions, and through conversation and gestures. Similar to how Johnson et al. (2017) found that using symbols provides opportunities for making powerful connections in dementia caregiving relationships, this study also found that connections can be facilitated through the use of symbolic interactions aided by multisensory stimulation, for example, dancing, singing, looking at photographs and watching films. These connections can have a particularly strong impact when facilitated between residents and care staff, who may not much prior knowledge on the person with dementia. Other work in this area has also found that sensory stimulation in dementia care can be a way of creating mutual relations between staff and residents (Lykkeslet et al., 2014).

The findings from this study found that music in particular prompted meaningful interactions between caregivers and participants with dementia. The residents’ desire to dance and sing could be interpreted as means of expressing their identities beyond verbal means. This can have important implications for people with advanced dementia, who may no longer have the capability to communicate verbally. For example, in one interview where a caregiver was speaking about a piece of music used in the sessions, the resident who had difficulties with verbal expression started humming the song. By doing this, she was able to engage in the conversation that was taking place.

The caregivers’ accounts of being touched by the ways in which the residents engaged with the music suggests a deeper connection to the individual was made. This is in agreement with other studies on music and dementia. McDermott et al. (2014) suggest that individual preference of music is preserved throughout the progression of dementia. Thus, the authors stress the importance of care personnel learning each resident’s musical history in order to promote musical and interpersonal connectedness, helping to maintain a sense of identity and quality of life (McDermott et al., 2014).

### A transactional model of narrative identity and relationships within SENSE-GARDEN

The findings from this study highlight the dynamic nature of interactions between not only people but also between person and environment. The ways in which the participants described their experiences inside the SENSE-GARDEN space reflects Deweyan philosophy in the sense that space was referred to as more than just being physical. For example, in recalling their experiences from inside the space, participants spoke about feeling an “energy” or feeling “transported”. This resonates with Peter Freund’s argument that “space is not merely a place in which social interaction occurs, it structures such interaction” (Freund, 2001, p. 694).

Furthermore, from the interviews, a clear interplay between past and present is distinguished. The idea that SENSE-GARDEN provokes reminiscence of past events and simultaneously prompts expression, communication and reflection in the present moment resonates with Deweyan philosophy. According to Dewey, there is no fixed self. Experience is temporally continuous, with past, present, and future being integrated with one another (Dewey, 1957). This is in line with more recent literature in this area. Edelman writes “Every perception, is some degree an act of creation, and every act of memory is to some degree an act of imagination” (Edelman, 2006, p. 123). Similarly, Rosenfield claims that “Recollection is a kind of perception … and every context will alter the nature of what is recalled” (Rosenfield, 1988, p. 89).

In the context of SENSE-GARDEN, the media contents trigger memories which are recalled and reflected upon in the present moment, loaded with new meanings and emotional connotations. For example, the participants often spoke of joy, mixed feelings or nostalgia when looking at old photographs. In this way, memories become stories that convey emotional importance (Wright-St Clair & Smythe, 2013). As Dewey writes, “the past is recalled not because of itself but because of what it adds to the present (Dewey, 1957, p. 2). The SENSE-GARDEN is arguably a means of recalling the past to create meaningful experiences in the present.

**Figure 5.**
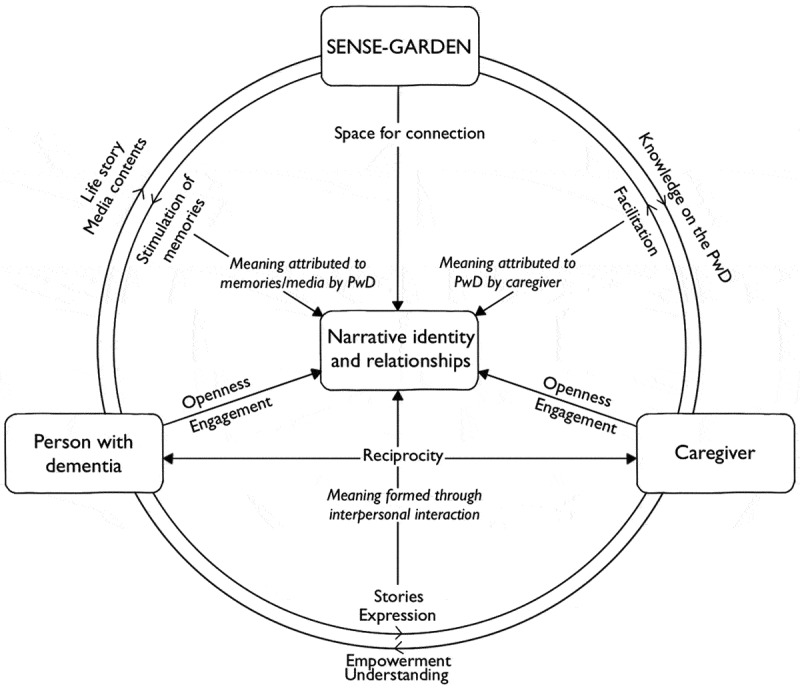
Transactional model of narrative identity and relationships facilitated through SENSE-GARDEN

In an attempt to make sense of these experiences within SENSE-GARDEN, a transactional model of how narrative identity and relationships are fostered through the use of the intervention has been created. The model, presented in Figure 5, considers the multiple factors that contribute to preserving and promoting narrative identity, of which instantiations will differ from person to person. For example, a person with dementia who lacks the ability to communicate verbally will need the opportunity for alternative methods of expression. In order to provide such an opportunity, the caregiver will need knowledge about the person with dementia’s life history and personal preferences in order to identify what kind of media contents could be useful in stimulating memories and prompting engagement and expression. Again, this media contents will differ from person to person, being dependent on the meaning that the person with dementia holds towards memories, events, and people in their lives. This personal knowledge can be hard to gain in the normal care setting during usual daily routines, especially when caring for residents with advanced dementia. However, through using the SENSE-GARDEN with the resident in a meaningful way (i.e., facilitating it in a way that encourages engagement from the resident), the caregiver has the opportunity to increase their understanding of the resident, which may benefit the caregiving relationship in terms of reciprocity and understanding. The caregiver can then plan and prepare future sessions using the new knowledge that they may have gained on the resident through previous sessions. This shows that flexibility in terms of individualization and facilitation is key in order for the intervention to be efficient.

The model also includes the factors that contribute to fostering relationships, such as reciprocity between the participant with dementia and the caregiver. However, this reciprocity is only achieved if the caregiver connects with the resident on a meaningful level, looking beyond the diagnosis of dementia. This meaningful connection will then, in turn, encourage the person with dementia to be more expressive and open with the caregiver. Similarly, Figueiredo et al. (2013) suggest that if caregivers provide opportunities to empower residents with dementia in long-term care, this may produce a “virtuous cycle” in which the well-being of the resident is improved, and, in turn, a sense of well-being and achievement is reinforced in caregivers.

The findings also suggest that meaning, which contributes to narrative identity and relationships, is generated in a constant flux between the SENSE-GARDEN environment and the participants inside the space. This resonates strongly with work conducted on transactional theory and occupational science. According to Dickie et al. (2006), who draw upon Deweyan philosophy to reflect on meaning-making in occupational science, meaning should be understood as flowing from the aesthetic, imaginative, creative and emotional modes of the transaction, not only in terms of the function of a transaction and its outcomes. Therefore, the influence of symbolic interactionism is also integrated into the model:

- *Meaning attributed to memories/media by PwD* refers to how the person with dementia holds meaning for the photographs, films and music within SENSE-GARDEN, and their associated memories.
- *Meaning attributed to PwD by caregiver* refers to caregiver’s perception and attitude towards the person with dementia, which are shaped through facilitating the sessions and learning more about the resident as an individual.
- *Meaning formed through interpersonal interaction* refers to how meaning is constructed through joint interaction between the person with dementia and the caregiver. This interaction is facilitated through the opportunity for expression, understanding, and reciprocity—all of which the participants experienced whilst using SENSE-GARDEN.

### Situating SENSE-GARDEN amongst similar technological solutions

The outcomes of this study draw similarities to studies on other kinds of reminiscence technologies. We found that SENSE-GARDEN can stimulate emotional experiences, help preserve narrative identity, and foster interpersonal relationships between people with dementia and their caregivers. Similarly, studies of the digital multimedia apps have found that they are useful for increasing a sense of identity, prompting conversation, and supporting social interaction amongst people with dementia (Critten & Kucirkova, 2019; Park et al., 2017; Samuelsson & Ekström, 2019; Subramaniam & Woods, 2016). Personalized music playlists, which require considerably less effort to configure, have also been shown to improve communication and evoke positive emotions amongst people with dementia (Huber et al., 2020; Long, 2017). Thus, with results being so similar to cheaper and more accessible technologies, one may question whether the cost of an expensive solution such as SENSE-GARDEN can be justified. One argument is that SENSE-GARDEN is not just a technological solution, but a space. Participants expressed that they enjoyed being inside the SENSE-GARDEN space. SENSE-GARDEN may also overcome barriers previously experienced in studies of other types of reminiscence technologies. For example, it could be easier to engage with media inside a space, compared to holding, using and/or viewing content on a touchscreen device (Critten & Kucirkova, 2019; Davison et al., 2016: Hashim et al., 2015). SENSE-GARDEN presents media on large walls, therefore not requiring the person with dementia to control any part of the technology. Nevertheless, further work should consider the cost-effectiveness of SENSE-GARDEN.

Furthermore, if this intervention is to be delivered on a long-term basis in the future, factors such as sustainability and scalability need to be considered. Issues such as time constraints amongst staff, perceived value of the intervention, and lack of motivation and energy amongst staff have recently been identified as barriers to implementing staff-led interventions into dementia care practice (Karrer et al., 2020; Kormelinck et al., 2020). With SENSE-GARDEN requiring a large time investment from staff in terms of preparing and facilitating sessions with individual residents, there is a risk that this intervention asks “too much” of staff members. In order to deliver this intervention at scale and ensure its continued use, multiple strategies on the use of SENSE-GARDEN could be explored. Alternative options may include: offering group sessions; using “generic” media content over personalized individual sessions; training additional personnel in the facility such as assistants and volunteers; creating pre-loaded media storage relating to specific themes, places, or eras that would be provided to the care facilities together with the SENSE-GARDEN solution. All these could be potential options for alleviating time pressure from what is already a fast-paced and busy environment. Whilst the strength of SENSE-GARDEN appears to be the meaningful interactions it facilitates between residents and caregivers, these interactions will be short-lived if the intervention cannot be scaled up and sustained in the long term. As Hirt et al. (2021) suggest in their study of nurse-led intervention in long-term dementia care, nurses should have the option of adjusting an intervention after it has been implemented. Whilst the options listed above may be useful to staff, it is ultimately the decision of nurses and other care professionals to integrate SENSE-GARDEN into their facilities in the way that they see best working for them.

### Limitations

This is a rather small study, based on a novel intervention. Therefore, the findings lack generalizability to other care homes. However, the knowledge generated from the findings may be applicable within a broader perspective of technology use beyond SENSE-GARDEN, as well as within the facilitation of meaningful activities in care.

Data collection was limited in two ways. First, the impact of the coronavirus pandemic hindered the way in which some interviews were conducted. Five interviews had to be conducted over the phone, which meant a lack of visual cues and expressions may have resulted in a less natural conversation. However, there is thought to be no significant differences between transcripts from telephone and face-to-face interviews (Sturges & Hanrahan, 2004). More importantly, the impact of the pandemic meant that five of the participants with dementia were not able to be interviewed. Second, there was a potential for bias during data collection. The interviews were conducted by the facilitators of the SENSE-GARDEN sessions. This would have most likely had an influence on how the participants chose to answer. Here, it is important to address researcher reflexivity. In being aware of and critical towards one’s own positionality within a study, a researcher should explicitly address the effect that this position may have on the research process and outcome (Berger, 2015; Dowling, 2006). Due to the relationships that formed between residents, informal caregivers, and facilitators over the course of the intervention, the data generated during the interviews may be less credible compared to having the interviews conducted by someone else with no connection to the intervention. However, given that the participants with dementia had moderate to severe dementia, we decided that the interviewer should be somebody who is familiar to the participants. Had another individual independent of the intervention conducted the interview, the conversational nature of the interviews would have been hindered and the participants may not have felt as comfortable. The advantage of having the facilitator conduct the interview was the fact that they could make the participants feel at ease, and also refer back to moments experienced together in the SENSE-GARDEN space as prompts during the interview. It is also important to note that the SENSE-GARDEN facilitators were not involved in the analysis of the transcripts or writing of this paper. The only interviewer who was involved with the analytic process was researcher and co-author LA who has a background in sociology and qualitative methods.

The analysis of data is also limited, mainly due to the fact that the transcripts were translated to English. Therefore, these transcripts lack the nuance of the quotes in their original language. However, the authors consist of one native English speaker, one native Norwegian speaker and two native Portuguese speakers. Together, the authors tried to ensure that the transcripts reflected what the participants were expressing in their original language.

Finally, the study of transactional relationships within the SENSE-GARDEN would have been enhanced with study of “in-the-moment” experiences. The analysis is based on reflections and interpretations of experiences already lived within the SENSE-GARDEN. Having included an observational element in situ would have provided further insight into the dynamic processes that take place within the intervention.

### Ethical considerations

Due to challenges concerning consent, participation, and safety, people with dementia are often excluded from many areas of research (Rivett, 2017). However, it is important that people with dementia are given opportunities to participate in research—especially individuals with moderate to advanced dementia. In the current study, careful considerations were made in the planning of the study to ensure the safety and well-being of the participants, as well as ensuring that their willingness to attend the SENSE-GARDEN sessions was respected. Information was given to residents and their informal caregivers before each step of the study by care professionals who had been involved with the planning and development of the intervention. Clear communication between researchers, care professionals, informal caregivers, and residents meant that all participants were kept well informed on the study and researchers were kept informed on any issues that had arisen. Most importantly, care professionals were able to continuously assess consent and willingness to participate by interacting with the residents before and after each SENSE-GARDEN session. Having built up a relationship over the 12–16 study period, the professionals were also able to assess whether or not the residents were willing to participate in an interview.

Another important consideration is the use of photographs in this article. The recording of photos and videos was included in the consent, along with the scientific and public dissemination of these materials. However, given the fact that consent was provided by proxy, it is important to address the ethical implications of using photographs. We felt the need to include the photos as a means of portraying the SENSE-GARDEN and its sessions in a way that words could not. However, the faces of the participants (as well as faces of individuals in photographs) have been blurred to respect the privacy of the participants.

Going forward, it is important to reflect upon the use of SENSE-GARDEN in the context of day-to-day use, outside of a research study, and what impact this may have on residents. An intervention that focuses so heavily on a person’s past memories is bound to evoke emotions that are not always positive. This has been the case in research of this nature, where including photographs of loved ones who have passed away in digital life stories has caused sadness amongst participants (Damianakis et al., 2010; Ryan et al., 2018). There may also be instances in which emotions are mixed. Whilst Swann (2013) acknowledges that the release any emotions can be good for the person with dementia, she also suggests that staff facilitating reminiscence activities should be sensitive to the emotions of residents, ready to offer comfort if needed, and ready to stop the activity if necessary.

### Implications for future research and practice

Future research on technology use in dementia care should adopt a holistic approach to considering not only the effect of the technology but also considering the situational context in which it is to be used. Technology design for dementia care, as Jiancaro, Jaglal and Mihailidis argue, is “deeply contextual” (2017: 576). This study has shown the benefit of integrating theoretical perspectives into exploring how technology may be used in care, particularly with regard to facilitating meaningful activities that promote narrative identity and relationships. Similar to Rosenberg and Nygård’s (2012) transactional approach to assistive technology use, our findings suggest that the use of technology for meaningful activities is complex and requires flexibility in order to be used efficiently. In the context of SENSE-GARDEN, possibilities for integrating partial automation into the creation and adaptation of the sessions is currently being explored. As one of the caregivers stated, it is difficult to prepare sessions manually. Furthermore, it is time-consuming to put together user profiles at the initial stage of preparing the sessions (approximately one hour per resident). If SENSE-GARDEN is to be used in everyday practice with multiple residents, there needs to be way of reducing the time taken to prepare sessions. Introducing this automated component may support caregivers in being able to prepare and facilitate sessions more easily and with less time constraints. Additionally, staff members should not be expected to have to manage any issues with the system themselves. Ensuring technical support is provided as needed by suppliers of the SENSE-GARDEN service, outside of a research context, is essential if the technology is to be used on a day-to-day basis.

Furthermore, the similarities between remarks made by caregivers in both Portugal and Norway, particularly regarding the lack of opportunities for residents to engage in meaningful activities, provokes an important question of whether there is still a serious lack of offer of such activities to people with moderate to advanced dementia on an international level. This study has shown how a technological solution such as SENSE-GARDEN can support care staff in providing meaningful activities, but more work needs to be done on how feasible it is to implement an intervention of this kind into a regular care routine within these environments. As mentioned by a member of staff in the present study, it is difficult to plan large numbers of SENSE-GARDEN sessions for one resident, especially when the contents of the session will need to be continuously adapted based on new information they receive from the resident. In order for an intervention such as SENSE-GARDEN to be used on a long-term basis, factors such as costs, time consumption, and staff resources and training, need to be considered.

## Conclusion

To conclude, there is promising potential for the use of technology for facilitating activities that may help construct narrative identities and promote interpersonal relationships within dementia care. Care residencies should incorporate the knowledge of residents into everyday activities in order to provide high-quality care, and the SENSE-GARDEN is an example of a tool that can be used to support this incorporation. A transactional perspective has illustrated the complex nature of the SENSE-GARDEN, and of person–environment interactions in general. In understanding the multiple factors that characterize the transactional relationships that take place through an intervention, implications for implementing and facilitating such intervention may be appreciated and assessed. These interactions—or transactions—need to be explored from a holistic approach. Whilst the technology offered by SENSE-GARDEN can be used for creating opportunities to engage with the life story of people with dementia, it is ultimately the relationships and interactions between people happening inside the space that gives meaning to the experience.

## Supplementary Material

Supplemental Material

## Data Availability

The data that support the findings of this study are openly available in Zenodo at https://doi.org/10.5281/zenodo.4081468
