# Supplementary material for: Supporting identity and relationships amongst people with dementia through the use of technology: a qualitative interview study
Source: Int J Qual Stud Health Well-being. 2021 May 6;16(1):1920349. doi: 10.1080/17482631.2021.1920349 (PMC8118425; doi:10.1080/17482631.2021.1920349)
Supplement: Supplemental Material [file ZQHW_A_1920349_SM0182.docx]

**Supplementary Material**

**Interview guide**

[adaptable to either single interview or group interview]

1. How would each of you describe your overall experience of using SENSE-GARDEN?
2. How did SENSE-GARDEN make you both feel?

- What was it about SENSE-GARDEN that made you feel that way?

1. What did you like about SENSE-GARDEN? Why?

- Prompts: ask about individual components of SENSE-GARDEN

1. Was there anything you did not like about SENSE-GARDEN? Why?
2. How did you feel about using the SENSE-GARDEN together?

- What did you talk about when using SENSE-GARDEN?
- Were there any specific memories/events you talked about?

1. Does using SENSE-GARDEN affect your ability to communicate with one another?

- In what ways?
- Why do you think this is?

1. How does using SENSE-GARDEN compare to everyday activities?
2. Would you use SENSE-GARDEN again?

- Why/why not?
- How often would you like to visit SENSE-GARDEN?

1. (If the participant was in the control group and stopped the visits after 12 weeks): How did you feel about having to stop the SENSE-GARDEN sessions?

- *(To the caregiver)* Have you noticed any effects or changes in [the participant with dementia] since they stopped the SENSE-GARDEN sessions?

1. Do either of you have any other comments that you would like to add?
